# KIAA1363 affects retinyl ester turnover in cultured murine and human hepatic stellate cells

**DOI:** 10.1016/j.jlr.2022.100173

**Published:** 2022-01-29

**Authors:** Carina Wagner, Victoria Hois, Annalena Eggeling, Lisa-Maria Pusch, Laura Pajed, Patrick Starlinger, Thierry Claudel, Michael Trauner, Robert Zimmermann, Ulrike Taschler, Achim Lass

**Affiliations:** 1 Institute of Molecular Biosciences, NAWI Graz, University of Graz, Graz, Austria; 2 Department of Surgery, General Hospital, Medical University of Vienna, Vienna, Austria; 3 Division of Hepatobiliary and Pancreatic Surgery, Department of Surgery, Mayo Clinic, Rochester, MN, USA; 4 Hans Popper Laboratory of Molecular Hepatology, Division of Gastroenterology and Hepatology, Department of Medicine III, Medical University of Vienna, Vienna, Austria; 5 BioTechMed-Graz, Graz, Austria; 6 Field of Excellence BioHealth, University of Graz, Graz, Austria

**Keywords:** KIAA1363, retinyl ester hydrolase, vitamin A, retinol, hepatic stellate cells, liver, JW480, α-SMA, alpha-smooth muscle actin, ATGL, adipose triglyceride lipase, cDNA, complementary DNA, ER, endoplasmic reticulum, FD, fluorescence detection, HSC, hepatic stellate cell, HSL, hormone-sensitive lipase, LAL, lysosomal acid lipase, LIPE, lipase E, NDUFS1, NADH:ubiquinone oxidoreductase core subunit S1, PC, phosphocholine, PNPLA2, patatin-like phospholipase domain-containing protein 2, PNPLA3, patatin-like phospholipase domain-containing protein 3, qPCR, quantitative PCR, R-BEL, (*R*)-bromoenol lactone, RE, retinyl ester, ROH, retinol, RP, retinyl palmitate

## Abstract

Large quantities of vitamin A are stored as retinyl esters (REs) in specialized liver cells, the hepatic stellate cells (HSCs). To date, the enzymes controlling RE degradation in HSCs are poorly understood. In this study, we identified KIAA1363 (also annotated as arylacetamide deacetylase 1 or neutral cholesterol ester hydrolase 1) as a novel RE hydrolase. We show that KIAA1363 is expressed in the liver, mainly in HSCs, and exhibits RE hydrolase activity at neutral pH. Accordingly, addition of the KIAA1363-specific inhibitor JW480 largely reduced RE hydrolase activity in lysates of cultured murine and human HSCs. Furthermore, cell fractionation experiments and confocal microscopy studies showed that KIAA1363 localizes to the endoplasmic reticulum. We demonstrate that overexpression of KIAA1363 in cells led to lower cellular RE content after a retinol loading period. Conversely, pharmacological inhibition or shRNA-mediated silencing of KIAA1363 expression in cultured murine and human HSCs attenuated RE degradation. Together, our data suggest that KIAA1363 affects vitamin A metabolism of HSCs by hydrolyzing REs at the endoplasmic reticulum, thereby counteracting retinol esterification and RE storage in lipid droplets.

In mammals, vitamin A is an essential fat-soluble micronutrient, required for the maintenance of life (1, 2). Large quantities of vitamin A are stored as retinyl esters (REs) in cytosolic lipid droplets of specialized liver cells, the hepatic stellate cells (HSCs) (3, 4). In times of nutritional undersupply, these hepatic RE stores are utilized to meet vitamin A requirements of the body (5).

The breakdown of RE stores requires the action of specific enzymes, so-called RE hydrolases. Several enzymes including lysosomal acid lipase (LAL), adipose triglyceride lipase (ATGL), hormone-sensitive lipase (HSL), and patatin-like phospholipase domain-containing protein 3 (PNPLA3) have been reported to be capable of hydrolyzing REs and to be expressed in HSCs (6, 7, 8, 9, 10). LAL has been shown in in vitro assays to be the main acid RE hydrolase in HSCs (10). However, LAL-deficient mice do not accumulate REs in the liver (9), and primary murine HSCs of these mice are capable of degrading REs (10). Similarly, ATGL-deficient and HSL-deficient mice show unchanged hepatic RE levels (6). Furthermore, isolated primary murine HSCs deficient of functional HSL or ATGL showed unchanged or partially reduced RE breakdown, respectively (6, 10). Human PNPLA3 exhibits hydrolase activity against retinyl palmitate (RP) (7), while this activity is reduced in the genetic variant I148M (11). Humans carrying the I148M variant are associated with increased hepatic RE levels (7, 12), lower plasma retinol (ROH) binding protein 4, and ROH levels (7, 13). Moreover, silencing of PNPLA3 in human HSCs resulted in reduced intracellular breakdown of REs and extracellular release of ROH (7). However, so far, any RE hydrolase activity has been demonstrated for the murine ortholog of PNPLA3 (6), and PNPLA3-deficient mice have not been reported to exhibit any changes in vitamin A homeostasis (14).

In a recent study (10), we evaluated the relative contribution of the neutral RE hydrolases ATGL, HSL, and PNPLA3 in human HSCs. In agreement with previous observations in respective knockout mice and cultivated HSCs (6, 7, 15), pharmacological inhibition of ATGL, PNPLA3, and HSL reduced RE hydrolase activity of human HSCs to some extent but did not impair RE degradation in cell experiments (10) suggesting that these neutral lipid hydrolases are not limiting for neutral RE degradation in HSCs. Thus, either the RE breakdown of HSCs is a highly redundant process and multiple enzymes are involved or the RE breakdown of HSCs is controlled by so far unknown RE hydrolase(s).

In this study, we identified KIAA1363 as a so far unrecognized neutral RE hydrolase. The enzyme is expressed in murine and human HSCs. Addition of the KIAA1363-specific inhibitor JW480 to lysates of murine and human HSCs largely reduced RE hydrolase activity. Furthermore, the presence of the inhibitor JW480 in the incubation media impaired RE degradation of murine and human HSCs. Similarly, also shRNA-mediated silencing of KIAA1363 expression attenuated RE degradation in human HSCs. Together, our data suggest that KIAA1363 affects RE turnover of HSCs.

## Materials and methods

### Material

Essentially FA-free BSA, ROH, RP, retinyl acetate, 1,2-dioleoyl-*sn*-glycero-3-phosphocholine (PC), Orlistat, JW480, and pLKO.1 puro vector DNA (Addgene; plasmid no.: 8453) were purchased from Sigma-Aldrich (St. Louis, MO). (*R*)-Bromoenol lactone (R-BEL) was purchased from Cayman Chemicals (Ann Arbor, MI). The HSL inhibitor NNC 0076-0000-0079 (76-0079) was a kind gift from Dr Christian Fledelius (Novo Nordisk A/S, Novo Nordisk Park, DK-2706 Måløv, Denmark). Human primary HSCs were provided by Patrick Starlinger, Department of Surgery, Medical University of Vienna, Vienna, Austria. The human HSC line LX-2 was used with the kind approval of Dr Scott L. Friedman (Icahn School of Medicine at Mount Sinai, New York).

### Methods

#### Cloning of recombinant HIS-tagged proteins

Total RNA was isolated from murine liver and human HSC LX-2 cells. RNA was digested with DNaseI and reverse transcribed into complementary DNA (cDNA) using LunaScript™ RT SuperMix kit (New England Biolabs, Inc, Ipswich, MA). The coding sequences of murine KIAA1363 (mKIAA1363; NM_178772.3) and human KIAA1363 (hKIAA1363; NM_020792.5) KIAA1363 were amplified by PCR from respective cDNAs using Q5 polymerase (New England Biolabs, Inc). The following primers were used: *mKiaa1363*_forward: 5′-GGA TCC AGG TCG TCA TGC GTC-3′, *mKiaa1363*_reverse: 5′-CTC GAG TCA CAG GTT TTG ATC-3′, *hKIAA1363*_forward: 5′-GGA TCC AGG TCG TCC TGT-3′, and *hKIAA1363*_reverse: 5′-CTC GAG TTA CAG GTT TTG ATC-3’. Coding sequences of mKIAA1363 and hKIAA1363 were subcloned into the pcDNA4/HisMaxC expression vector (Invitrogen GmbH, Lofer, Germany) to generate expression vectors encoding N-terminal HIS-tagged mKIAA1363 or hKIAA1363, respectively. In brief, PCR products and vector were digested with BamHI and XhoI, ligated using T4 DNA ligase, and transformed into competent *Escherichia coli* C2987 cells (all reagents from New England Biolabs, Inc). Plasmid DNA from transformed *E. coli* C2987 cells was isolated using QIAprep Spin Minikit (Qiagen, Hilden, Germany). Plasmid DNA sequences were validated by commercial DNA sequencing (Microsynth AG, Balgach, Switzerland). Large-scale isolation of plasmid DNA was performed using NucleoBond® Xtra Plasmid Purification Kit (Macherey-Nagel, Düren, Germany). A control pcDNA4/HisMaxC vector encoding β-galactosidase (LacZ) was provided by the manufacturer (Invitrogen GmbH). The coding sequence of mKIAA1363 was subcloned into pEGFP-C1 vector to generate an N-terminal GFP-tagged recombinant protein. Following primers were used for subcloning: GFP_*mKiaa1363*_forward: 5′-AAC TCG AGG AAG GTC GTC ATG C-3′ and GFP_*mKiaa1363*_reverse: 5′-GTG GAT CCT CAC AGG TTT TGA TC-3’.

#### Expression of recombinant proteins using Expi293F™ expression system

Suspension-adapted human embryonic kidney cells (Expi293F™, Gibco®; Invitrogen GmbH) were cultivated in Expi293F™ expression medium (Gibco®; Invitrogen GmbH) under constant shaking (125 rpm) at 37°C, humified atmosphere, and 7% CO_2_. Cells were diluted to a final density of 3.0 × 10^6^ cells/ml. Transfection was performed using plasmid DNA encoding the respective HIS-tagged recombinant proteins or LacZ (as control) and the transfection reagent ExpiFectamine™ 293 reagent (Gibco®; Invitrogen GmbH), according to manufacturer's instructions. One day post-transfection, ExpiFectamine™ 293 Transfection Enhancer 1 and ExpiFectamine™ 293 Transfection Enhancer 2 were added to the cell suspensions. Two days post-transfection, cells were harvested by centrifugation.

#### Preparation of cell lysates and determination of protein content

Cell lysates were prepared from cell suspensions in solution A (0.25 M sucrose, 1 mM EDTA, 1 mM DTT, 20 μg/ml leupeptin, 2 μg/ml antipain, and 1 μg/ml pepstatin, pH 7.0) by sonication (2 × 10 s, amplitude 15%; Sonoplus ultrasonic homogenizer HD3100; Bandelin electronic GmbH & Co KG, Berlin, Germany). Nuclei and unbroken cells were removed by centrifugation at 1,000 *g* for 10 min at 4°C. Lysates were stored at −20°C until further use. Protein concentrations were determined by Bio-Rad protein assay according to manufacturer's instructions using BSA as standard (Bio-Rad, Hercules, CA).

#### Preparation of cell organelle-enriched fractions

Expi293F™ cells were transfected with plasmids encoding N-terminal HIS-tagged mKIAA1363 or N-terminal HIS-tagged LacZ as control as described previously. Cells were harvested 48 h post-transfection and lysed in fractionation buffer (50 mM NaCl, 0.25 M sucrose, 1 mM Tris-HCl, 20 μg/ml leupeptin, 2 μg/ml antipain, and 1 μg/ml pepstatin, pH 7.4) by passing cells through a 26G needle (20 repetitions). Nuclei and unbroken cells were removed by centrifugation at 1,000 *g* for 10 min at 4°C (= 1,000 *g* supernatant). Mitochondria-enriched fractions were obtained by centrifugation of the 1,000 *g* supernatant at 9,000 *g* for 15 min at 4°C (= 9,000 *g* pellet). Microsome-enriched fractions were obtained by centrifugation of the 9,000 *g* supernatant at 20,000 *g* for 1 h at 4°C (= 20,000 *g* pellet). The resulting 20,000 *g* supernatant represents the cytosol-enriched fraction.

#### Cultivation and expression of recombinant mKIAA1363 in COS-7 cells

COS-7 cells were cultured in DMEM (4.5 g/l glucose; Gibco®, Invitrogen GmbH), supplemented with 10% FCS and antibiotics at 37°C under humidified atmosphere and 7% CO_2_. COS-7 cells were seeded in 12-well plates at a density of 0.2 × 10^6^ cells/ml. The following day, cells were transfected with plasmid DNA encoding for N-terminal HIS-tagged mKIAA1363 or LacZ (as control) using Metafectene® (Biontex, Munich, Germany), according to manufacturer's instructions. One day post-transfection, cells were used for experiments.

#### Silencing of hKIAA1363 in human HSC LX-2 cells

For silencing of hKIAA1363 in LX-2 cells, the pLKO.1-TRC vector system (Addgene, Watertown, MA) was used (16). A short hairpin (sh) siRNA sequence targeting hKIAA1363 (LX-2 sh: 5′-GCT CAA ACT ACA AGC TTT AAT-3′) and a scrambled (scr) control sequence (LX-2 scr: 5′-GGC CTG AAC TCC ATA CTG ATT-3′) were designed using siRNA Wizard Software 3.1 (InvivoGen, San Diego, CA) and inserted in the following oligo sequences: forward oligo: 5′-CCG G—21 bp sense—CTC GAG—21 bp antisense—TTT TTG-3′ and reverse oligo: 5′-AAT TCA AAA A—21 bp sense—CTC GAG—21 bp antisense-3’. Annealing of oligos (100 pmol per oligo) was performed in a thermocycler by cooling down from 95°C to room temperature at a rate of 1°C per 3 min. pLKO.1 vector DNA was digested with AgeI and EcoRI (New England Biolabs, Inc) and ligated with the annealed oligos using T4 ligase. pLKO.1 vector DNA containing the LX-2 sh or LX-2 scr shRNA sequence was transformed into competent *E. coli* C2987 cells (New England Biolabs, Inc). Plasmid DNA from transformed *E. coli* C2987 cells was isolated using QIAprep Spin Minikit (Qiagen). Plasmid DNA sequences were verified by commercial DNA sequencing (Microsynth AG).

The recombinant pLKO.1-TRC vector was transfected together with psPAX2 packing and pMD2.G envelope plasmid in HEK293T cells to generate lentiviral particles. In brief, HEK293T cells were seeded in a 6-well plate at a density of 0.85 × 10^6^ cells. The next day, cells were preincubated with 25 μM chloroquine diphosphate for 5 h and then incubated with polyethylenimine/plasmid DNA mix (15 μg polyethylenimine and 1.6 μg per plasmid DNA per 6-well) for 24 h. Two days post-transfection, supernatant containing lentiviral particles was collected and used for infection of target cells. LX-2 cells were seeded in 6-well plates at a density of 0.2 × 10^6^ cells. The following day, LX-2 cells were incubated with 8 μg/ml polybrene and infected with 1 ml of the virus suspension for 24 h. Cells were centrifuged at 300 *g* for 1 h at room temperature. One day postinfection, 1 μg/μl puromycin was added to cultivation media to select for infected cells. Infected cells were subsequently cultivated in DMEM containing 1% FCS, antibiotics, and puromycin for further experiments.

#### Diet studies

C57Bl/6J mice were housed on a regular light-dark cycle (14 h light, 10 h dark) at 22 ± 1°C in a specific pathogen-free environment and had ad libitum excess to food and water. Mice were kept either on a standard laboratory chow diet (R/M-H Extrudate V1126-027, containing ∼15,000 IU/kg vitamin A, Ssniff Spezialdiaeten GmbH, Soest, Germany), a vitamin A-deficient diet for 9 weeks (10 mm pellets E15311-14, containing <120 IU/kg vitamin A, Sniff), or a high fat/vitamin A-excess diet for 3 weeks (EF R/M pellets, E15744-034; containing 45 kJ % fat, 175 mg/kg CHOL, and 83,000 IU/kg β-carotene). Overnight fasted (∼16 h) male mice were anesthetized with isoflurane and euthanized by cervical dislocation. Tissues were dissected, flash frozen in liquid nitrogen, and stored at −80°C until further use. All animal experiments were approved by the Austrian Federal Ministry for Science, Research, and Economy (protocol number GZ: 39/9/75 ex 2017/18) and conducted in compliance with the Council of Europe Convention (ETS 123).

#### Isolation of primary HSCs by collagenase perfusion and cultivation by selective detachment

Primary human HSCs were isolated from liver resections for metastasis of colon-rectal cancer, approved by the Ethic Committees of Medical University of Vienna (EK Nr: 2032/2013) as described (17). HSCs were cultured in DMEM (4.5 g/l glucose; Gibco®, Invitrogen GmbH) containing 10% FCS (Sigma-Aldrich) and 100 μg/ml primocin. Primary human HSCs between passage 3 and 6 were used for experiments.

Primary HSCs of WT mice (male/female, 2 months of age) were isolated as described previously by Blomhoff *et al.* (18) with some modifications. Briefly, mice were anesthetized, and the abdomen was surgically opened. The liver was perfused via the portal vein with Krebs-Henseleit buffer (without Ca^2+^ and SO_4_^2−^) for 5 min, followed by a perfusion with Krebs-Henseleit buffer containing 0.2 mg/ml collagenase type II (Worthington Biochemical Corporation, Lakewood, NJ), 2% BSA, and 0.1 mM CaCl_2_ for 10 min. Afterward, the liver was excised, minced, and the cell suspension was passed through a gauze, followed by filtration through a 70 μm cell strainer. Hepatocytes were separated from nonparenchymal cells by centrifugation at 50 *g* for 3 min at 4°C. Supernatant containing the nonparenchymal cell fraction was centrifuged at 900 *g* for 5 min at 4°C. Pelleted nonparenchymal cells were suspended in DMEM (4.5 g/l glucose; Gibco®, Invitrogen GmbH) containing 10% FCS (Sigma-Aldrich) and 100 μg/ml primocin. Nonparenchymal cells were plated and cultivated at 37°C in humidified air at 80% saturation and 5% CO_2_. Cultures of primary HSCs were obtained by selective detachment for 12–14 days. After 2–3 days in culture, cells were trypsinized and replated. This resulted in the removal of nonstellate cells from the culture. After 10 days of culture, more than 90% of cells stained positive for alpha-smooth muscle actin (α-SMA) (6). Noncultivated primary HSCs were isolated by isopycnic centrifugation using OptiPrep™ self-forming density gradient solutions (Axis-Shield PoC AS, Rodeløkka, Norway) as described previously (19). Briefly, nonparenchymal cell suspension (pool of five WT mice) was adjusted to a density of 24% iodixanol, overlaid with 11.5%, 8.4%, and 0% iodixanol in Krebs-Henseleit buffer containing 1.25 mM CaCl_2_ and 1.2 mM Na_2_SO_4_. After centrifugation at 1,400 *g* for 20 min at 4°C, HSCs were collected at the 8.4/0% iodixanol interphase.

#### ROH loading of cells and pulse-chase experiments

ROH loading and pulse-chase cell experiments were performed as previously described (10). Briefly, LX-2 and COS-7 cells were incubated for 20–24 h in DMEM (4.5 g/l glucose) supplemented with 1% or 10% FCS, respectively, 40 μM ROH (stock solution in ethanol), and 300 μM oleic acid (4 mM stock solution in PBS complexed to essentially FA-free BSA in a ratio of 3:1, M:M). Primary HSCs were incubated for 20–24 h in DMEM (4.5 g/l glucose) supplemented with 10% FCS, 20 μM ROH, and 100 μM oleic acid. After ROH loading, medium was replaced with serum-free DMEM (1 g/l glucose) supplemented with 2% FA-free BSA for 8 h (serum-starvation period). In some cases, medium contained the pharmacological inhibitors Orlistat (20 μM), JW480 (10 μM), or DMSO as solvent control.

#### Extraction and quantification of retinoids by HPLC-fluorescence detection

Extraction and quantification of retinoids by HPLC-fluorescence detection (FD) was performed as previously described with some modifications (10). In brief, cells were washed twice with PBS, and retinoids were extracted twice with 1 ml *n*-hexane:2-propanol (3:2; v/v) for 10 min under constant shaking at room temperature. Organic phases were combined and dried in a speed-vac (Labconco, Kansas City, MO). For retinoid analysis by HPLC-FD, retinoid extracts were dissolved in methanol:toluene (1:1; v/v) and separated on a YMC-Pro C18 column (150 × 4.6 mm, S-3 μl, 12 nm, YMC Europe GmbH, Dinslaken, Germany) using a gradient solvent system (flow, 2 ml/min; gradient, 1–2 min 100% methanol, 2–4.2 min 60%/40% methanol/toluene, and 4.2–6 min 100% methanol). Fluorescence was detected at excitation 325 nm/emission 490 nm. The HPLC consisted of a Waters e2695 separation module, a column oven (at 25°C), and a Waters 2475 fluorescence detector (Waters Corporation, Milford, MA). Area under the peak was standardized against known amounts of reference compounds using Empower 3 chromatography data software (Waters Corporation). For normalization of cellular retinoid content, cells were dissolved in NaOH/SDS, and cell protein was determined with Pierce™ BCA Protein Assay Kit (Thermo Fisher Scientific).

#### Measurement of in vitro RE hydrolase activity

Determination of in vitro RE hydrolase activity was performed as previously described with some modifications (10). Briefly, 100 μl cell or tissue lysates (50–100 μg protein), containing pharmacological inhibitors, 76-0079 (20 μM), Orlistat (20 μM), JW480 (10 μM), or DMSO (solvent control), were incubated with 100 μl RP (300 μM, stock solution in toluene containing 1 mM butylhydroxytoluene) as substrate for 1 h at 37°C under constant shaking in a water bath. Substrate was emulsified with PC (300 μM) in potassium phosphate buffer (100 mM, pH 7.5) or potassium acetate buffer (100 mM, pH 4.9) containing 4% FA-free BSA. Substrate blank incubation was performed with solution A. After incubation, 1 ml *n*-hexane and 200 μl ethanol containing 3.67 μM retinyl acetate as internal standard were added, and samples were vigorously vortexed. Phase separation was obtained by centrifugation at 5,000 *g* at 4°C for 10 min. Upper organic phase was collected and dried in a speed-vac. Retinoid extracts were dissolved in 100 μl methanol:toluene (1:1, v/v) and analyzed by HPLC-FD.

#### Isolation of total RNA and analysis of gene expression by quantitative PCR

Isolation of RNA, DNA digestion, cDNA synthesis, and quantitative PCR (qPCR) were performed as previously described (10, 20). Following primers were used for gene expression analysis: *patatin-like* *phospholipase* *domain-containing* *protein 2 (PNPLA2)*_forward: 5′-GTG TCA GAC GGC GAG AAT G-3′; *PNPLA2*_reverse: 5′-TGG AGG GAG GGA GGG ATG-3′; *lipase E (LIPE)*_forward: 5′-CTG CAT AAG GGA TGC TTC TAT GG-3′; *LIPE*_reverse: 5′-GCC TGT CTC GTT GCG TTT G-3′; *PNPLA3*_forward: 5′-GGC ATC TCT CTT ACC AGA GTG T-3′; *PNPLA3*_reverse: 5′-GGC ATC CAC GAC TTC GTC TTT-3′; *ABHD5*_forward: 5′-ACA GAC CTG TCT ATG CTT TTG AC-3′; *ABHD5*_reverse: 5′-AGG GCA CAT CTC CAC TCT TCA-3′; *G6Pase*_forward: 5′-CCT CCT CAG CCT ATG TCT GC-3’; *G6Pase*_reverse: 5′-AAC ATC GGA GTG ACC TTT GG-3’; *α-SMA*_forward: 5′-TCA GGG AGT AAT GGT TGG AAT G-3’; *α-SMA*_reverse: 5′-TCG GCA GTA GTC ACG AAG GAA-3’; *mKiaa1363*_forward: 5′-AAG GTC TTC TCC GAA AGT GAA GG-3’; *mKiaa1363*_reverse: 5′-CCT CCG TGG ATA TAG ATG ACG C-3’; *Lrat*_forward: 5′-ACA AGG AAC GCA CTC AGA AG-3’; *Lrat*_reverse: 5′-GTC TAG GTG ATT GAC GAG GAT G-3’; *CycloB*_forward: 5′-GGC TCC GTC GTC TTC CTT TT-3’; *CycloB*_reverse: 5′-ACT CGT CCT ACA GAT TCA TCT CC-3′; *36B4*_forward: 5′-GCT TCA TTG TGG GAG CAG ACA-3′, and *36B4*_reverse: 5′-CAT GGT GTT CTT GCC CAT CAG-3’. Target gene expression was calculated by the ΔΔCT method. Expression of ribosomal housekeeping genes *CycloB* or *36B4* was used for normalization.

#### Analysis of protein expression by immunoblotting

Analysis of protein expression by immunoblotting was performed as previously described (10). Proteins of cell lysates (20–40 μg) were dissolved in SDS sample buffer, separated by 10% or 12.5% SDS-PAGE, and transferred onto a polyvinylidene fluoride membrane (Carl Roth GmbH, Karlsruhe, Germany). The membrane was blocked with 10% nonfat dry milk and incubated with the following primary antibodies: anti-ATGL, anti-HSL, anti-GAPDH, and anti-calnexin from Cell Signaling Technology (Danvers, MA; 2138S/ATGL, 4107S/HSL, 2118S/GAPDH, 2679S/calnexin), anti-6x HIS tag, and anti-NADH:ubiquinone oxidoreductase core subunit S1 (NDUFS1) from Abcam (Cambridge, England; ab18184/6x HIS-tag®, ab157221/NDUFS1), anti-α-SMA from Thermo Fisher Scientific (Waltham, MA; PA5-22251/α-SMA), and anti-KIAA1363 from Invitrogen GmbH (PA5-50285/NCEH1 = KIAA1363), respectively. For detection, membranes were incubated with horseradish peroxidase-labeled secondary antibodies specific for respective primary antibody. Bands were visualized using the ECL plus Western blotting Detection Reagent (Thermo Fisher Scientific) and ChemiDoc Touch Imaging System (Bio-Rad).

#### Microscopy

For confocal microscopy, COS-7 cells were seeded in 8-well chamber slides and transfected with plasmid DNA encoding for N-terminal GFP-tagged mKIAA1363 using Metafectene® (Biontex) according to manufacturer's instructions. In some cases, cells were cotransfected with an endoplasmic reticulum (ER) marker protein DsRed, which encodes for DsRed fused N-terminally with the ER targeting sequence of calreticulin and C-terminally with the ER retention sequence (KDEL) (21). Microscopy was performed 48 h after transfection on a Leica SP5 confocal microscope (Leica Microsystems, Inc, Germany) using a 63×, numerical aperture 1.4 HCX PL APO oil immersion objective. Excitation and emission wavelengths were set to properties of the respective fluorescent tags.

#### Statistical analyses

Data are presented as mean + SD. Statistically significant differences were determined by Student's unpaired *t*-test (two-tailed). Group differences were considered statistically significant for *P* < 0.05 (∗), *P* < 0.01 (∗∗), and *P* < 0.001 (∗∗∗).

## Results

### mKIAA1363 and hKIAA1363 exhibit RE hydrolase activity in vitro

**Fig. 1:**
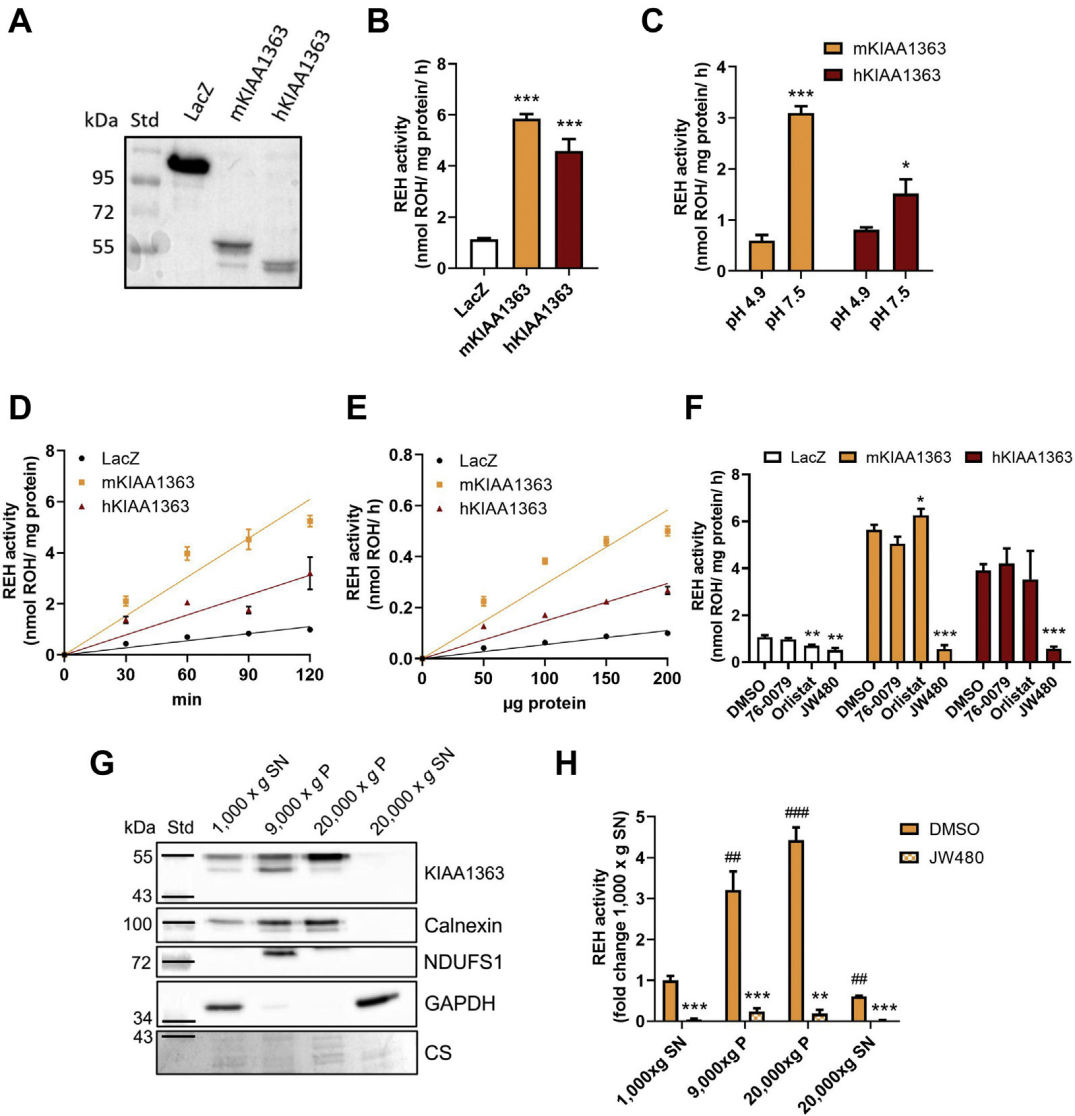
KIAA1363 exhibits RE hydrolase activity at neutral pH. Expi293F™ cells were transfected with plasmids encoding N-terminal HIS-tagged mKIAA1363 and hKIAA1363 or LacZ as control. Subsequently, lysates were prepared. A: Expression of HIS-tagged proteins was analyzed by Western blotting. B–F and H: For in vitro REH activity assay, cell lysates containing recombinant proteins were incubated with RP (300 μM) as substrate for 1 h. Substrate was emulsified with PC (300 μM) in potassium phosphate buffer (100 mM, pH 7.5) or potassium acetate buffer (100 mM, pH 4.9), as indicated, and 2% FA-free BSA were added. Retinoids were *n*-hexane extracted, and ROH content was analyzed by HPLC-FD. D: Cell lysates were incubated with substrate for various time points as indicated. E: Increasing amounts of cell lysates, as indicated, were incubated with substrate. F: Small-molecule inhibitors or DMSO as solvent control were added to the cell lysates: 76-0079 (20 μM), Orlistat (20 μM), or JW480 (10 μM). G and H: Cell lysates containing mKIAA1363 were fractionated by differential centrifugation: 1,000 *g* supernatant (SN), 9,000 *g* pellet (P, mitochondria-enriched/microsome-enriched fraction), 20,000 *g* P (microsome-enriched fraction), and 20,000 *g* SN (cytoplasmic-enriched fraction). G: Protein content of mKIAA1363, calnexin, GAPDH, and NDUFS1 was analyzed by Western blotting. Coomassie stain (CS) was used as loading control. H: Cell fractions were incubated with substrate containing DMSO (solvent control) or the small-molecule inhibitor, JW480 (10 μM). Data are mean + SD and representative for three independent experiments (n = 3). Statistically significant differences were determined by Student's unpaired *t*-test (two-tailed: ∗*P* < 0.01; ∗∗*P* < 0.01; ∗∗∗*P* < 0.001 between groups; ##*P* < 0.01; ###*P* < 0.001 between cell fractions).

In a search for novel RE degrading enzymes with structural homology to HSL, we identified KIAA1363 to be capable of hydrolyzing REs. This finding prompted us to investigate the functional role of mKIAA1363 and hKIAA1363 in RE metabolism in more detail. Expression of N-terminal HIS-tagged KIAA1363 orthologs was analyzed by Western blotting (Fig. 1A), showing a double band at ∼56 kDa for mKIAA1363 and at ∼46 kDa for hKIAA1363, presumably representing proteins differing in the degree of glycosylation (20) (Fig. 1A). Subsequently, cell lysates were used to determine RE hydrolase activity. Cell lysates containing mKIAA1363 or hKIAA1363 showed 5-fold and 4-fold increased RE hydrolase activity, respectively, as compared with LacZ-containing control lysates (Fig. 1B). RE hydrolase activity of lysates containing the murine and human recombinant proteins was much higher at neutral (pH 7.5) than acidic pH (pH 4.9) (Fig. 1C), confirming that KIAA1363 is most active at neutral pH (21). To validate in vitro RE hydrolase activities of mKIAA1363 and hKIAA1363, we varied incubation periods and protein concentrations, revealing that RE hydrolase activity of mKIAA1363 and hKIAA1363 increased in a time-dependent (Fig. 1D) and dose-dependent (Fig. 1E) manner. Since KIAA1363 belongs to the family of serine hydrolases and shows structural homology to HSL (22), we employed Orlistat, a general serine hydrolase inhibitor, and the small-molecule inhibitor 76-0079, known to inhibit HSL. Interestingly, the addition of 76-0079 or Orlistat did not reduce in vitro RE hydrolase activity of mKIAA1363 or hKIAA1363 (Fig. 1F). However, the addition of JW480, a KIAA1363-specific inhibitor (23), decreased in vitro RE hydrolase activity of lysates containing the mKIAA1363 or hKIAA1363 by 90% and 86%, respectively (Fig. 1F). Together, results of in vitro activity assays demonstrate that the murine and human orthologs of KIAA1363 exhibit neutral RE hydrolase activity.

KIAA1363 has been reported to localize to the ER (24). To validate the subcellular localization of KIAA1363, we performed cell fractionation experiments using Expi293F™ cell lysates containing N-terminal HIS-tagged mKIAA1363. We then subjected respective cell fractions to Western blotting. Protein bands for mKIAA1363 were more intense in the mitochondria/microsome-enriched 9,000 *g* pellet (which showed intense bands for marker proteins NDUFS1 and calnexin, respectively) and in the microsome-enriched 20,000 *g* pellet (with an intense band for the marker protein calnexin, Fig. 1G). No mKIAA1363 protein band was detected in the cytosol-enriched 20,000 *g* supernatant, which contained the marker protein GAPDH (Fig. 1G). Respective cell fractions were subsequently used for in vitro activity assay. RE hydrolase activities of the 9,000 *g* and 20,000 *g* pellet fractions (enriched in mKIAA1363 protein) were 3-fold and 4-fold higher, whereas that of the 20,000 *g* supernatant was lower as compared with the 1,000 *g* supernatant (Fig. 1H). Addition of JW480 virtually blunted RE hydrolase activities of all fractions (Fig. 1H) indicating that most of the RE hydrolase activities detected derived from KIAA1363. To visualize microsomal localization of KIAA1363, we expressed a hydrolytic active recombinant GFP-fusion protein (GFP-mKIAA1363, supplemental Fig. S1A) in COS-7 cells and performed laser-scanning live cell imaging. Cells were cotransfected with DsRed as ER marker. Signals obtained from GFP-mKIAA1363 showed a network-like distribution throughout the cell (supplemental Fig. S1B), which was similar to that obtained for the ER marker protein DsRed. A merge of the two signals showed a high degree of overlap, consistent with an ER localization of KIAA1363 (supplemental Fig. S1B).

### Expression of KIAA1363 lowers RE levels of COS-7 cells

**Fig. 2:**
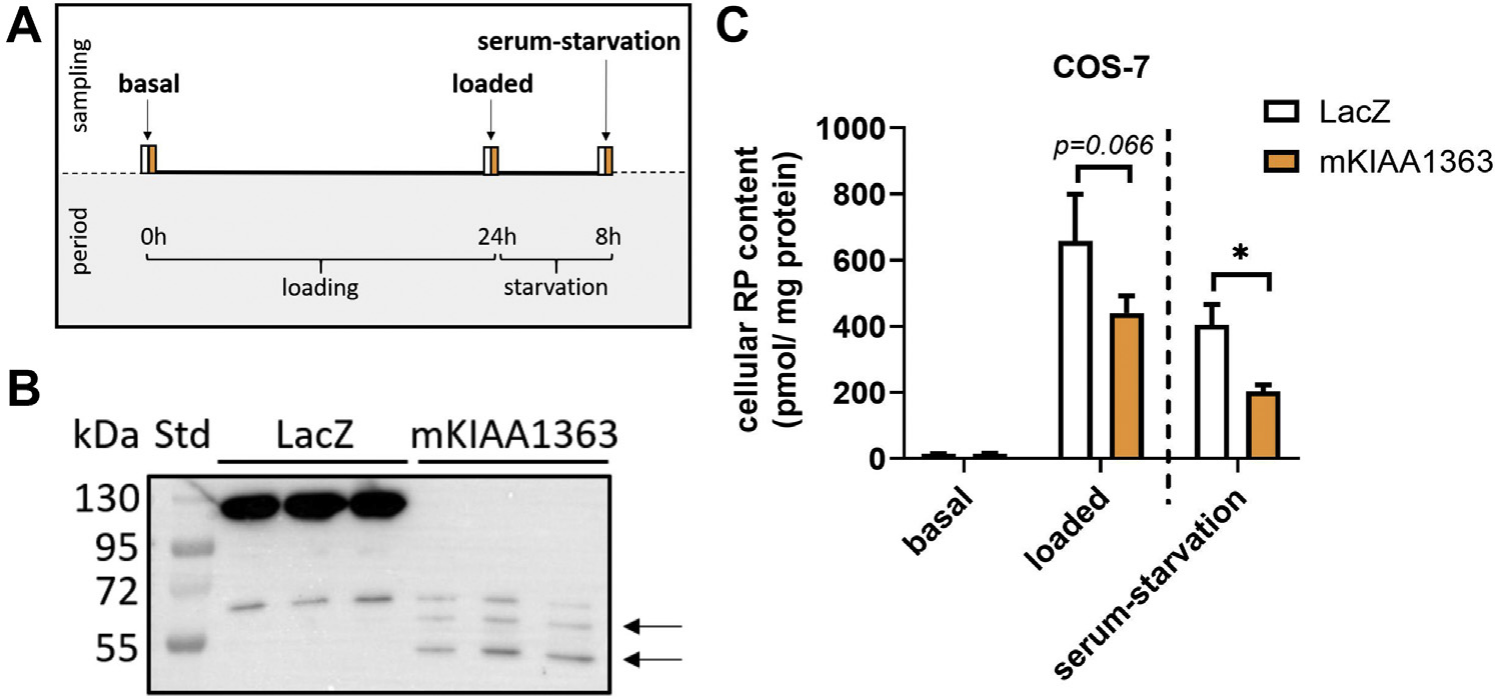
Expression of KIAA1363 lowers RE levels in COS-7 cells. A: Schematic representation of pulse-chase cell experiment. B, C: COS-7 cells were seeded in 12-well plates and transfected with plasmids encoding HIS-tagged mKIAA1363 or LacZ (control). B: Protein expression levels of HIS-tagged recombinant proteins were analyzed by Western blotting (arrows indicate bands corresponding to KIAA1363 protein). C: COS-7 cells expressing LacZ or mKIAA1363 were cultured with DMEM supplemented with 10% FCS and antibiotics (=basal). Then, cells were incubated in DMEM containing 10% FCS, oleic acid (300 μM), and ROH (40 μM) for 24 h (=loaded/pulse). After the loading period, cells were incubated with DMEM low glucose (1 g/l) containing 2% FA-free BSA for 8 h (=serum starvation/chase). Lipids were extracted with *n*-hexane:2-propanol (3:2, v/v). RP content was determined by HPLC-FD and normalized to milligram cell protein. Data are mean + SD and representative for two independent experiments (n = 3). Statistically significant differences were determined by Student's unpaired *t*-test (two-tailed: ∗*P* < 0.05).

To investigate whether KIAA1363 affects cellular RE levels, we transfected COS-7 cells with plasmids encoding mKIAA1363 or LacZ (as control) and performed pulse-chase experiments. One day post-transfection, COS-7 cells were loaded with ROH and oleic acid to promote RE formation (lipid loading/pulse period). After 24 h, cells were serum starved to induce RE degradation (serum starvation/chase period). At all indicated time points, cellular retinoid levels were determined (for schematic representation of experiment, see Fig. 2A). Expression of recombinant HIS-tagged proteins was confirmed by Western blotting analysis (Fig. 2B). Under basal conditions, mKIAA1363 or LacZ-expressing cells contained low amounts of RP (Fig. 2C, see “basal”). ROH loading led to increased cellular RP levels in LacZ-expressing and mKIAA1363-expressing cells. Yet, mKIAA1363-expressing cells exhibited tentatively lower cellular RP levels as compared with LacZ-expressing cells (Fig. 2C, see “loaded”). In the chase period, cellular RP content of LacZ-expressing and mKIAA1363-expressing COS-7 cells decreased, whereby mKIAA1363-expressing cells contained ∼50% lower RP levels (Fig. 2C, see “serum starvation”). The lower cellular RP levels in KIAA1363-expressing cells during lipid loading and serum-starvation periods indicate that KIAA1363 counteracts RP formation at the ER rather than mobilizing REs from existing LDs. This is consistent with the observation that KIAA1363 localizes to the ER.

### KIAA1363 is expressed in HSCs

**Fig. 3:**
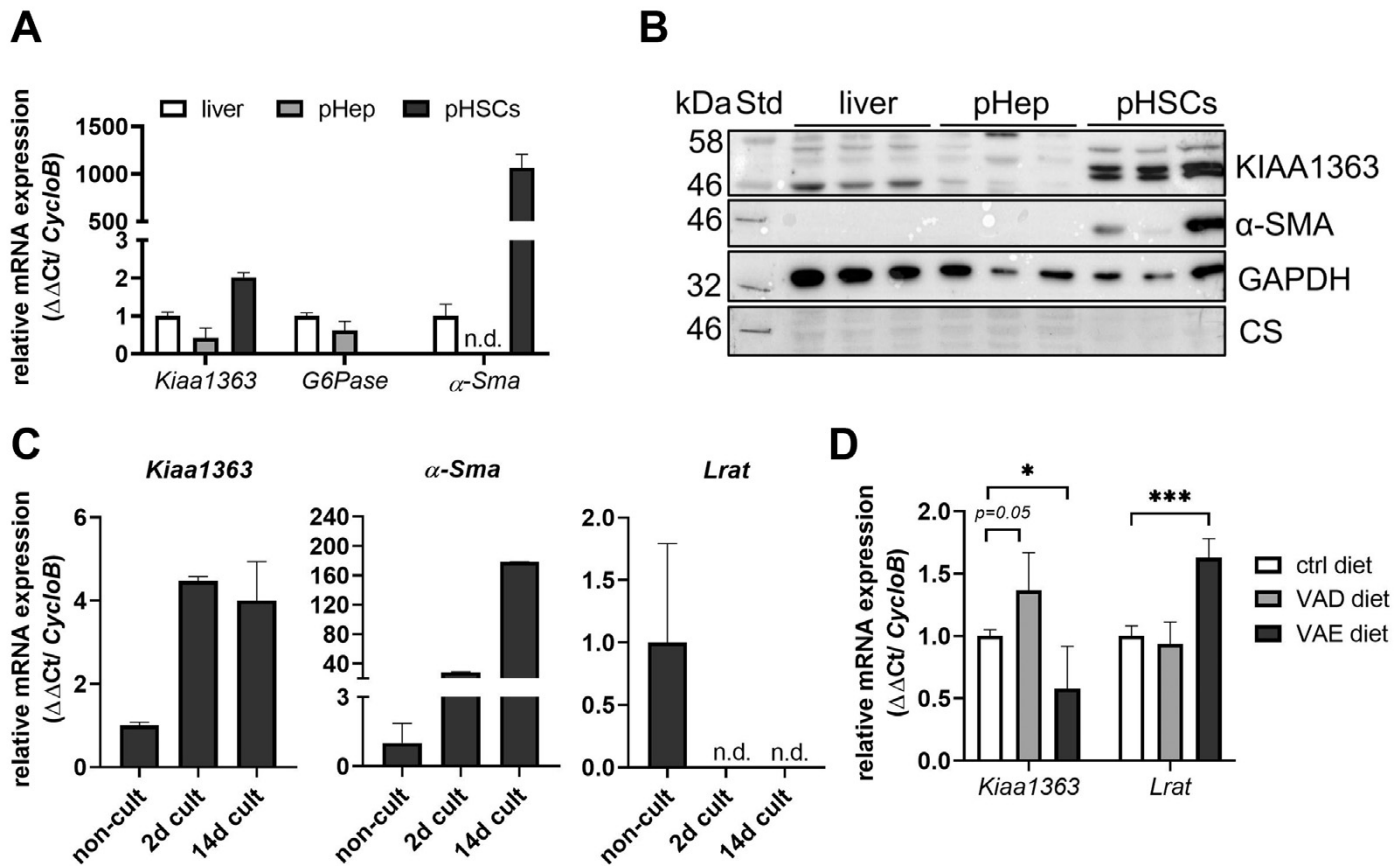
KIAA1363 exhibits low expression in whole liver but is highly expressed in primary murine HSCs. Primary hepatocytes (pHep) and HSCs (pHSC) were isolated from WT mice by liver perfusion, collagenase digestion, and centrifugation. NPC fraction was cultivated for 2–14 days in DMEM supplemented with 10% FCS and ROH (5 μM) to obtain pure pHSC cultures. Noncultivated primary HSCs were isolated by differential centrifugation of the NPC fraction. A: RNA was isolated from liver, pHep, and cultivated pHSCs. mRNA expression levels of *Kiaa1363*, *G6Pase*, and *α-Sma* were determined by qPCR. mRNA expression levels were normalized to housekeeping gene *CycloB*. B: Lysates from liver (1,000 *g* supernatant) as well as pHep and pHSCs were prepared, and protein expression levels of KIAA1363, α-SMA, and GAPDH were analyzed by Western blot analysis. C: RNA was isolated from noncultivated, 2 days cultivated, and 14 days cultivated HSCs, and mRNA expression levels of *Kiaa1363*, *α-Sma*, and *Lrat* were determined by qPCR. Noncultivated HSCs were isolated from a pool of five separate preparations. D: RNA of mice fed a standard chow diet, vitamin A-deficient (VAD) diet, or vitamin A-excess (VAE) diet were isolated (n = 4). mRNA levels of *Kiaa1363* and *Lrat* were determined by qPCR. Data are mean + SD and representative for two independent experiments (n = 2–3). Statistically significant differences were determined by Student's unpaired *t*-test (two-tailed: ∗*P* < 0.05, ∗∗∗*P* < 0.001). n.d., nondetectable; NPC, nonparenchymal.

Since retinoids are mainly stored in liver, we next investigated the expression pattern of KIAA1363 in total liver and primary murine hepatocytes and HSCs by qPCR and Western blotting. While primary hepatocytes of sufficient purity were obtained by centrifugation, HSCs were purified by selective detachment and cultivation for 2 weeks. Purity of primary hepatocytes and HSCs was confirmed by high mRNA expression of the hepatocyte marker *glucose-6-phosphate* (*G6Pase*) and the stellate cell marker *α-Sma*, respectively (Fig. 3A). Analysis of mRNA levels showed highest expression of *Kiaa1363* in primary HSCs compared with primary hepatocytes and total liver (Fig. 3A). Accordingly, Western blot analysis showed protein bands at around 50 kDa for KIAA1363 in primary HSCs, whereas KIAA1363-specific bands were not detectable in total liver lysates and primary hepatocytes (Fig. 3B). α-SMA expression levels were used as stellate cell marker and Coomassie stain as well as GAPDH protein levels as loading control, respectively (Fig. 3B). Since cultivation of HSCs on plastic surfaces leads to their activation (as evident from high α-SMA expression), we further investigated mRNA expression of *Kiaa1363* in freshly isolated noncultivated “quiescent” HSCs and compared it with its expression after 2 and 14 days of cultivation. Expression of *Kiaa1363* was detectable in noncultivated quiescent HSCs, and its expression was ∼4-fold increased after 2 and 14 days of cultivation (Fig. 3C, left). As expected, *α-Sma* expression was low in noncultivated “quiescent” HSCs and increased upon the duration of cultivation (Fig. 3C, middle). Vice versa, *Lrat* was expressed in noncultivated “quiescent” HSCs but not detectable after 2 and 14 days of cultivation (Fig. 3C, right). Interestingly, in activated HSCs, which are known to lose their vitamin A stores (25, 26), *Lrat* and *Kiaa1363* mRNA expression was inversely regulated. Since *Lrat* expression in liver is known to be vitamin A sensitive (27), we explored the possibility whether also *Kiaa1363* mRNA would be differently expressed in liver of mice when fed different vitamin A feeding regimes. qPCR measurements of hepatic mRNA for *Lrat* and *Kiaa1363* revealed that upon vitamin A-excess diet, hepatic *Kiaa1363* expression was decreased, whereas that of *Lrat* increased (Fig. 3D). Conversely, upon vitamin A-deficient diet, hepatic mRNA of *Kiaa1363* was tentatively higher than of mice on standard chow diet, while no change in expression levels was observed for *Lrat* expression (Fig. 3D). This observation further bolstered our hypothesis that KIAA1363 might play a role in hepatic RE degradation.

### Pharmacological inhibition of KIAA1363 impairs RE degradation in primary murine HSCs

**Fig. 4:**
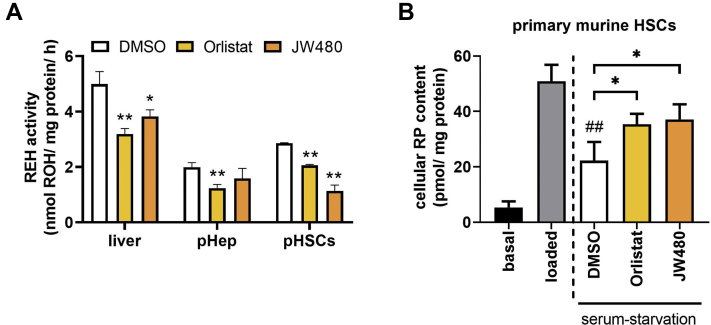
Pharmacological inhibition of KIAA1363 lowers RE degradation in primary murine HSCs upon serum starvation. A: For in vitro REH activity assay, lysates (1,000 *g* supernatant) of liver as well as pHep and pHSCs were incubated with RP (300 μM) as substrate. Substrate was emulsified with PC (300 μM) in potassium phosphate buffer (100 mM, pH 7.5) containing 2% FA-free BSA. Small-molecule inhibitors or DMSO as solvent control were added to cell lysates as indicated: Orlistat (20 μM) and JW480 (10 μM). Lipids were *n*-hexane extracted, and ROH content was analyzed by HPLC-FD. B: Primary murine HSCs were cultured in DMEM supplemented with 10% FCS and antibiotics (=basal). Then, cells were incubated in DMEM containing 10% FCS, ROH (20 μM), and oleic acid (100 μM) for 24 h (=loaded/pulse). Cells were incubated with DMEM low glucose (1 g/l) containing 2% FA-free BSA for 8 h (=serum starvation/chase). Lipids were extracted with *n*-hexane:2-propanol (3:2, v/v). RP content was determined by HPLC-FD and normalized to milligram cell protein. Data are mean + SD and representative for three independent experiments (n = 3). Statistically significant differences were determined by Student's unpaired *t*-test (two-tailed: ∗*P* < 0.05; ∗∗*P* < 0.01, between control and inhibitors; ##*P* < 0.01 between “loaded” and “serum-starvation DMSO”).

To assess the contribution of KIAA1363-dependent RE hydrolase activity in murine liver as well as of different liver cell types, we employed the KIAA1363-specific inhibitor JW480 and for comparison the inhibitor Orlistat, known to inhibit several serine hydrolases such as ATGL but not KIAA1363 (28, 29). Addition of Orlistat decreased in vitro RE hydrolase activity in lysates of liver, primary hepatocytes, and primary HSCs by 36%, 38%, and 29% (Fig. 4A), respectively. Addition of JW480 led to a 20% and 60% reduction of in vitro RE hydrolase activity in lysates of liver and primary HSCs, whereas no effect was observed in primary hepatocytes (Fig. 4A). This suggests that KIAA1363 plays a role in the turnover of REs in HSCs.

To examine the role of KIAA1363 in RE turnover of HSCs, we isolated primary murine HSCs and performed pulse-chase cell experiments. In these experiments, we loaded primary HSCs with ROH and oleic acid and added the inhibitors Orlistat or JW460 during the serum-starvation period (for schematic representation of experiment, see Fig. 2A). At all indicated time points, we determined cellular RP content. Under basal culturing conditions, primary murine HSCs contained low amounts of cellular RP (Fig. 4B, see “basal”). Lipid loading with ROH and oleic acid increased cellular RP levels many-fold (Fig. 4B, see “loaded”). Serum starvation led to a 50% decrease of cellular RP levels (Fig. 4B, see “serum-starvation DMSO”). In the presence of Orlistat or JW480, only 30% and 27% of RPs were degraded during the serum-starvation period causing a 1.6-fold increase of RP levels as compared with DMSO control (Fig. 4B, compare “serum-starvation DMSO,” “Orlistat,” and “JW480”). Together, results of expression analyses, activity assays, and cell experiments clearly show that (i) KIAA1363 is expressed in murine HSCs, (ii) KIAA1363 contributes to the majority of in vitro RE hydrolase activity in primary murine HSCs, and (iii) both KIAA1363-dependent and KIAA1363-independent pathways affect RE metabolism in cultured primary murine HSCs.

### Pharmacological inhibition of KIAA1363 impairs RE degradation upon serum starvation in LX-2 cells and primary human HSCs

**Fig. 5:**
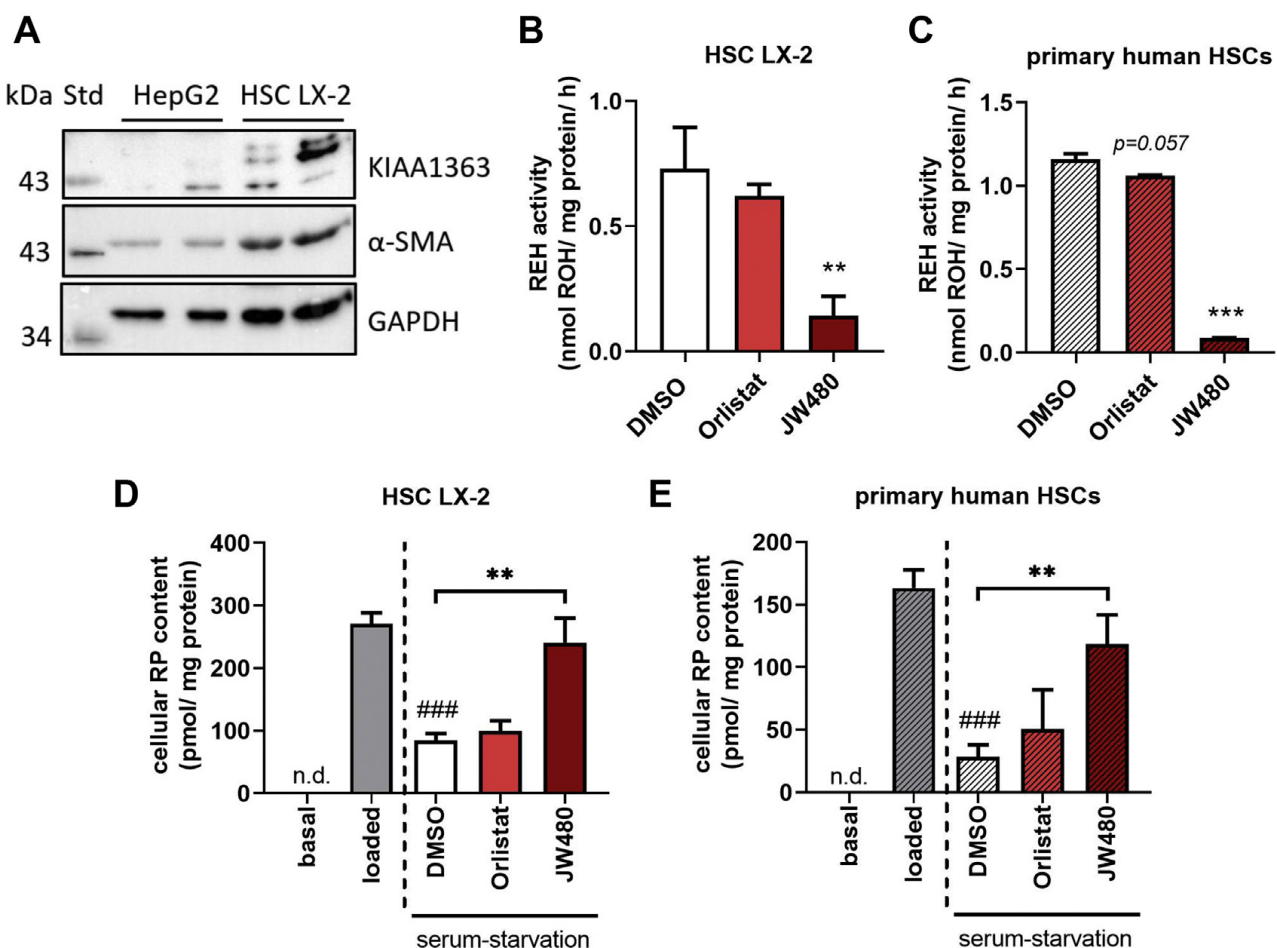
Pharmacological inhibition of hKIAA1363 impairs RE degradation in LX-2 cells and primary human HSCs upon serum starvation. A: For in vitro REH activity assay, cell lysates (1,000 *g* supernatant) from HepG2 and LX-2 cells were prepared, and protein expression was determined by Western blotting. Cell lysates (1,000 *g* supernatant) of (B) HSC LX-2 and (C) primary human HSCs were incubated with RP (300 μM) as substrate. RP was emulsified with PC (300 μM) in potassium phosphate buffer (100 mM, pH 7.5) containing 2% FA-free BSA. Small-molecule inhibitors or DMSO as solvent control were added to cell lysates as indicated: Orlistat (20 μM) and JW480 (10 μM). Lipids were *n*-hexane extracted, and ROH content was analyzed by HPLC-FD. D: LX-2 and (E) primary human HSCs were seeded in 12-well plates and cultured in DMEM supplemented with 1% or 10% FCS and antibiotics (=basal), respectively. Then, LX-2 and primary human HSCs were incubated with DMEM containing 1% or 10% FCS, 40 μM or 20 μM ROH, and 300 μM or 100 μM oleic acid for 24 h (=loaded/pulse), respectively. Cells were incubated with DMEM low glucose (1 g/l) containing 2% FA-free BSA for 8 h (=serum starvation/chase). Serum-starvation media contained small-molecule inhibitors or DMSO as solvent control as indicated: Orlistat (20 μM) and JW480 (10 μM). Cellular neutral lipids were extracted with *n*-hexane:2-propanol (3:2, v/v). RP content was determined by HPLC-FD and normalized to milligram cell protein. Data are mean + SD and representative for two to three independent experiments (n = 3). Statistically significant differences were determined by Student's unpaired *t*-test (two-tailed: ∗∗*P* < 0.01; ∗∗∗*P* < 0.001, between control and inhibitors; ###*P* < 0.001 between “loaded” and “serum-starvation DMSO”). n.d., nondetectable.

Since we observed that KIAA1363 contributes to RE hydrolysis in murine HSCs, we next asked whether this was also conserved in human HSCs. We used the immortalized human HSC cell line LX-2 and isolated and cultured primary human HSCs. We first investigated the protein expression levels of KIAA1363 in LX-2 cells and for comparison in the human hepatocyte HepG2 cell line. Western blotting confirmed protein expression of KIAA1363 in LX-2 cells, whereas no band was detectable in HepG2 cells (Fig. 5A). To dissect the contribution of KIAA1363 and other serine hydrolases capable of hydrolyzing REs, we again performed activity assays using the KIAA1363-specific inhibitor JW480 and the general serine hydrolase inhibitor Orlistat. In LX-2 cell lysates, the addition of Orlistat decreased in vitro RE hydrolase activity by 15%, whereas JW480 decreased hydrolytic activity by 80% (Fig. 5B). Using lysates of primary human HSCs as source of enzymatic activity, the addition of JW480 led to an even more pronounced reduction of in vitro RE hydrolase activity (by 92%), whereas Orlistat exerted virtually no effect (Fig. 5C).

We next assessed the contribution of KIAA1363 in RE turnover in human HSCs in pulse-chase experiments. We loaded LX-2 cells with ROH and oleic acid (=loading/pulse) and then serum-starved LX-2 cells (=serum starvation/chase, for schematic representation of experiment, see Fig. 2A). In some cases, cells were incubated with different pharmacological inhibitors during serum starvation. Under basal culturing conditions (=basal), LX-2 cells contained nondetectable amounts of RP. ROH loading led to accumulation of cellular RP in LX-2 cells, which decreased by 70% after the serum-starvation period, indicative for degradation of cellular REs (Fig. 5D, compare “basal,” “loaded,” and “serum-starvation DMSO”). LX-2 cells treated with Orlistat during serum starvation showed similar cellular RE levels as DMSO-treated control cells (Fig. 5D, compare “serum-starvation DMSO” and “Orlistat”). In contrast, the presence of JW480 during the serum-starvation period completely abolished RE degradation (Fig. 5D, compare “loaded,” “serum-starvation DMSO,” and “JW480”).

Similarly, as observed in experiments using human LX-2 cells, isolated and cultured primary human HSCs did not contain detectable amounts of RP (Fig. 5E, see “basal”). Loading of primary human HSCs with ROH and oleic acid led to an accumulation of cellular RP, which after a serum-starvation period decreased by 83% (Fig. 5E, compare “loaded” and “serum-starvation DMSO”). While the presence of Orlistat during serum starvation had no effect on cellular RP degradation, JW480 led to 4-fold higher cellular RP levels as compared with DMSO control (Fig. 5E, compare “serum-starvation DMSO,” “Orlistat,” and “JW480”).

Results of this inhibitor study indicate that KIAA1363 contributes to a large extent to RE hydrolase activity and affects RE metabolism in human HSCs.

### Knockdown of KIAA1363 in LX-2 cells attenuates RE degradation upon serum starvation

**Fig. 6:**
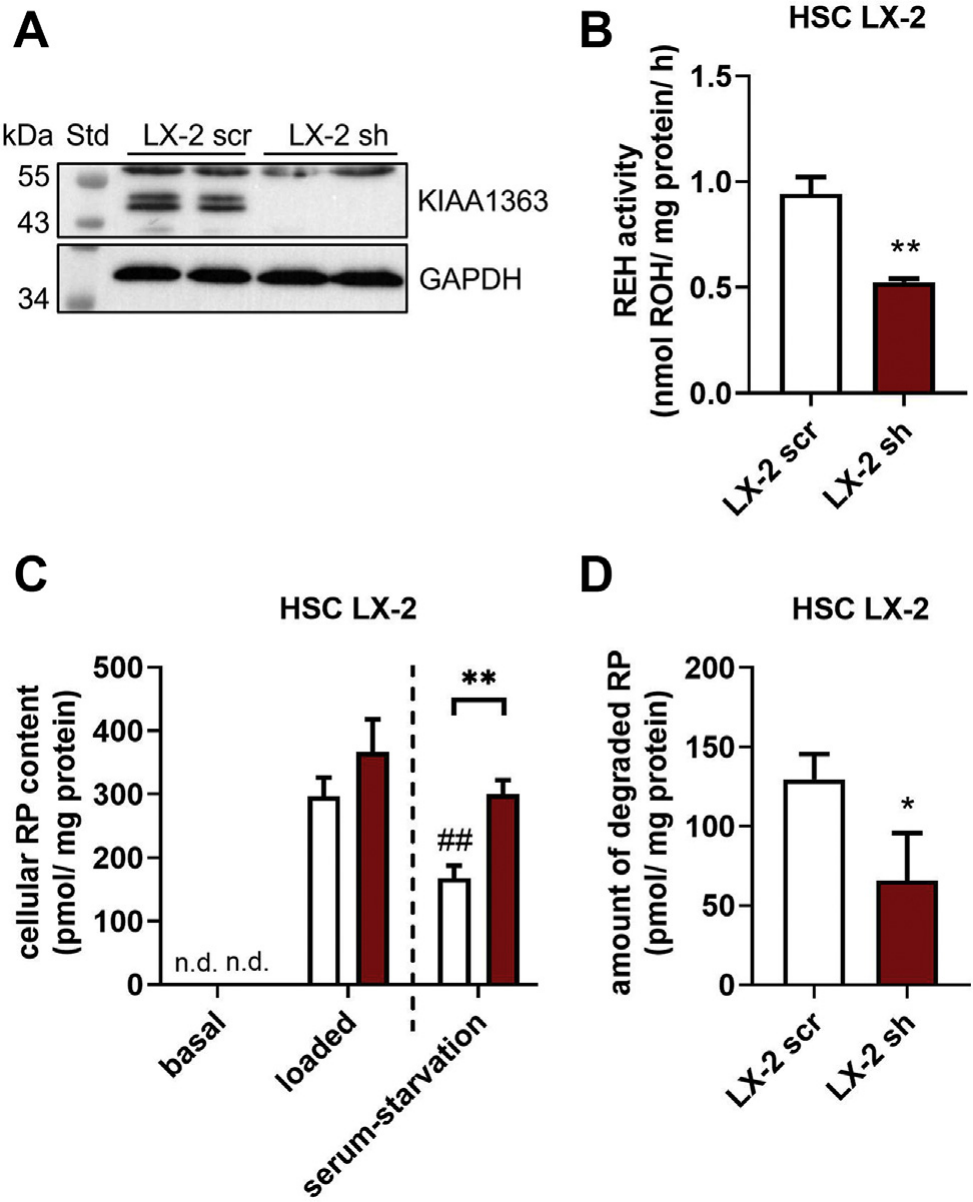
Knockdown of KIAA1363 in HSC LX-2 cells impairs RE degradation upon serum starvation. KIAA1363 knockdown in LX-2 cells was achieved by lentiviral particles, encoding sh sequence targeting hKIAA1363 (LX-2 sh) or a scrambled sh sequence (LX-2 scr) as control. A: Protein expression of KIAA1363 and GAPDH was determined by Western blot analysis. B: For in vitro REH activity assay, lysates (1,000 *g* supernatant) of LX-2 scr and LX-2 sh cells were prepared and incubated with RP (300 μM) as substrate. RP was emulsified with PC (300 μM) in potassium phosphate buffer (100 mM, pH 7.5) containing 2% FA-free BSA. Lipids were *n*-hexane extracted, and ROH content was analyzed by HPLC-FD. C: LX-2 scr and LX-2 sh cells were seeded in 12-well plates and cultured in DMEM supplemented with 1% FCS and antibiotics (=basal). Then, cells were incubated with DMEM containing 1% FCS, ROH (40 μM), and oleic acid (300 μM) for 24 h (=loaded/pulse). LX-2 cells were incubated with DMEM low glucose (1 g/l) containing 2% FA-free BSA for 8 h (=serum starvation/chase). Cellular lipids were extracted with *n*-hexane:2-propanol (3:2, v/v). RP content was determined by HPLC-FD and normalized to milligram cell protein. D: The amounts of degraded RP were calculated by subtracting the amounts after “serum starvation” from that of “loaded” cells. Data are mean + SD and representative for three independent experiments (n = 3). Statistically significant differences were determined by Student's unpaired *t*-test (two-tailed: ∗*P* < 0.05; ∗∗*P* < 0.01; ∗∗∗*P* < 0.001, ##*P* < 0.01 between “loaded” and “serum starvation”). n.d., nondetectable.

To confirm pharmacological experiments with a genetic approach, we next generated LX-2 cells with stable knockdown of KIAA1363 by siRNA. This knockdown was achieved by lentiviral particles using the pLKO.1-TRC vector system encoding a sh sequence targeting hKIAA1363 (LX-2 sh) and a scrambled sequence (LX-2 scr) as control. Western blot analysis showed that expression of KIAA1363 was not detectable in LX-2 sh cells as compared with control LX-2 scr cells, confirming a successful knockdown of hKIAA1363 expression (Fig. 6A). As expected, in vitro RE hydrolase activity of LX-2 sh cell lysates was reduced by 45% (Fig. 6B).

Since the knockdown of KIAA1363 might have led to compensatory changes in the expression of other RE hydrolases, such as *PNPLA2* (*PNPLA2* = *ATGL*), its coactivator comparative gene identification-58 (also known as α/β-hydrolase domain-containing protein 5, short ABHD5), LIPE (LIPE = *HSL*), and *PNPLA3*, we performed qPCR and Western blot analysis. Interestingly, we did not observe any changes in the gene or protein expression levels in LX-2 sh cells as compared with control cells (supplemental Fig. S2A, B). Protein expression level of the activation marker α-SMA was also unchanged in both cell lines (supplemental Fig. S2B). To analyze the contribution of ATGL, PNPLA3, and HSL in in vitro RE hydrolysis of LX-2 sh cells in detail, we employed several enzyme inhibitors. The addition of R-BEL, known to inhibit human ATGL and PNPLA3, as well as Orlistat, reduced in vitro RE hydrolase activity of both cell lines between 15% and 30%. No changes in RE hydrolase activity were observed when 76-0079, a specific HSL inhibitor, was applied (supplemental Fig. S2C). Together, data show that neither ATGL, HSL, nor PNPLA3 were compensatory regulated upon KIAA1363 knockdown. Furthermore, since the addition of Orlistat and R-BEL to lysates of LX-2 cells showed some reduction in in vitro RE hydrolase activity, data suggest that other RE hydrolases including ATGL and/or PNPLA3 are expressed and contribute to some extent to overall RE hydrolase activity.

To investigate the effect of KIAA1363 knockdown on RE turnover in LX-2 cells, we performed pulse-chase experiments and measured cellular RE content (for schematic representation of experiment, see Fig. 2A). Upon ROH loading, cellular RP content of LX-2 scr and LX-2 sh cells increased many-fold, showing tentatively higher RP levels in LX-2 sh cells (Fig. 6C, compare “basal” and “loaded”). While cellular RP levels decreased in LX-2 scr cells upon serum starvation by 50%, those of LX-2 sh cells remained much higher (Fig. 6C, compare “loaded” and “serum starvation”). Calculation of the amounts of degraded RP during serum starvation showed that when KIAA1363 expression was knocked down in HSC LX-2 cells (LX-2 sh), the amounts of degraded cellular RPs were much lesser than that of control cells (LX-2 scr, see Fig. 6D). Together, results of the pulse-chase experiment clearly demonstrate that silencing of KIAA1363 expression impairs RE degradation of LX-2 cells upon serum starvation, indicating that KIAA1363 plays an important role in RE turnover in human HSCs.

## Discussion

The molecular mechanisms controlling vitamin A homeostasis are incompletely understood. The body's largest vitamin A reserves are stored as REs in specialized liver cells, the HSCs. This store is maintained to meet the vitamin A requirements of the body. To date, the enzyme(s) controlling the degradation of REs in HSCs are unknown. In this study, we characterized the functional role of KIAA1363 as novel RE hydrolase. We show that KIAA1363 acts as neutral RE hydrolase in HSCs, as evident from the following observations: (i) KIAA1363 is expressed in HSCs. (ii) Cell lysates containing recombinant KIAA1363 exhibited increased RE hydrolase activity in in vitro activity assays that was inhibited by the KIAA1363-specific small-molecule inhibitor, JW480. (iii) RE hydrolase activity of cell lysates containing recombinant KIAA1363 increased in a time-dependent and dose-dependent manner. (iv) Expression, pharmacological inhibition, as well as silencing of KIAA1363 affected cellular RE levels.

KIAA1363, annotated as arylacetamide deacetylase-like 1 or neutral cholesterol ester hydrolase 1, belongs to the arylacetamide deacetylase protein family. It harbors an α/β-hydrolase fold and is therefore a member of the α/β-hydrolase superfamily. Within this superfamily, KIAA1363 shares high structural homologies to HSL (22). Amino acid sequence analysis of murine, rat, and human HSL and hKIAA1363 revealed that predicted α-helices and β-sheets of respective proteins are well conserved, even though they only share 22% sequence identity. The main structural homologies are found in the lipid binding and catalytic domain, containing the HG dipeptide and the active serine in a G-X-S-X-G motif. However, KIAA1363 is lacking the regulatory domain and the N-terminal region of HSL. Instead, KIAA1363 contains an N-terminal putative transmembrane domain consisting of a 23 hydrophobic amino acid stretch and a putative C-terminal diarginine retention/retrieving signal. These structural differences are in accordance with a different cellular localization, as KIAA1363 but not HSL localizes to ER membranes (22, 30). In agreement with structural features and previous reports (22, 24), we observed enriched KIAA1363 protein levels and KIAA1363-dependent RE hydrolase activity in microsomal fractions of cells. Furthermore, live cell imaging using GFP-tagged KIAA1363 confirmed ER localization.

The cellular localization of KIAA1363 suggests that it acts at the ER to promote RE mobilization. This may be facilitated by counteracting the esterification of ROH by acyltransferases such as lecithin:ROH acyltransferase, and as a consequence, lesser REs are channeled into lipid droplets. Since a certain portion of lipid droplets remains physically associated with ER membranes, enzymes of the ER may have access to the lipid moiety of lipid droplets. Thus, KIAA1363 may also degrade REs stored in lipid droplets that are in close contact to ER membranes. Similar to KIAA1363, esterase-22, an ER-localized RE hydrolase (mainly expressed in hepatocytes) has been shown to attenuate the formation of cellular RE stores in pulse-chase experiments in COS-7 cells (18).

KIAA1363 has been shown to be expressed in heart, adrenal glands, kidney, brain, and macrophages, whereas no expression was detectable in liver (22). Furthermore, KIAA1363 expression is induced by retinoic acid receptor-related orphan receptor α-agonist (31) but is not a target of protein kinase A (24). In our study, we found that hepatic KIAA1363 mRNA expression is lower in mice fed a vitamin A-deficient diet, suggesting that KIAA1363 expression might be regulated by the availability of vitamin A to meet requirements of the body. However, under standard chow diet, KIAA1363 protein was not detectable in lysates of total liver or of primary hepatocytes but in isolated primary HSCs. Furthermore, KIAA1363 expression was induced upon HSC activation. Since HSCs account for only ∼5% of total liver (3) and KIAA1363 expression is rather low in quiescent HSCs, which might explain why in total liver, protein expression of KIAA1363 was below detection limit. Furthermore, using the KIAA1363-specific inhibitor, JW480, we observed a pronounced inhibition of RE hydrolase activity in primary HSCs but not in primary hepatocytes. This pattern was consistent with the expression pattern of KIAA1363 in liver cell types and indicates that KIAA1363 contributes to total RE hydrolase activity in HSCs. RE hydrolase activity of murine HSCs was also inhibited by Orlistat (30% reduction) indicating that other serine hydrolases contribute to RE hydrolase activity of HSCs. Orlistat was originally developed to inhibit gastric and pancreatic lipase (32); later it was found to lack selectivity and thus is used as a general serine hydrolase inhibitor (29). Orlistat also inhibits RE hydrolase activity of ATGL and HSL (29). Since these lipases are known to be expressed in HSCs (6, 8), it thus might well be that these proteins are at least partly responsible for the Orlistat-inhibitable RE hydrolase activity of HSCs. Furthermore, these observations are in line with previous reports showing that ATGL and HSL are not limiting for RE degradation (6, 10, 33). In addition, and as suggested by in vitro activity assays using primary HSCs, we observed in pulse-chase experiments using the human HSC LX-2 cell line and primary murine and human HSCs that JW480 impaired the breakdown of cellular RE stores. This effect was even more pronounced in the human HSC LX-2 cell line as compared with murine HSCs. Interestingly, Orlistat exerted no effect on cellular RE stores in pulse-chase experiments with human HSC LX-2 cells, whereas the KIAA1363-specific inhibitor, JW480, completely abolished RE degradation in the chase period. This suggests that the RE hydrolase activity of KIAA1363 is not only conserved in mouse and human HSCs but may even play a more pronounced role in RE turnover of human HSCs. This conclusion is further supported by results of gene silencing experiments of KIAA1363 in LX-2 cells, where we observed reduced in vitro RE hydrolase activity and impaired cellular RE degradation in pulse-chase experiments after serum starvation. However, our pulse-chase cell experiments involved the cultivation of HSCs, which leads to their activation and increased KIAA1363 expression. Thus, KIAA1363 may play a more prominent role under conditions when HSCs get activated and its expression is induced.

In addition to the observation of this study that KIAA1363 affects RE turnover of HSCs, previous reports on ATGL-deficient and HSL-deficient mice have shown that any of these enzymes plays a critical role in vitamin A homeostasis as none of these mouse models develop signs of vitamin A deficiency (e.g., reduced plasma ROH levels or changes in hepatic RE levels, respectively) (6, 14, 15). So far, the vitamin A status of KIAA1363 knockout mice has not been investigated. Thus, future studies are required to explore the functional role of KIAA1363 in vitamin A homeostasis in, for example, a mouse model lacking functional KIAA1363.

The literature suggests different enzymatic functions for KIAA1363: (i) mKIAA1363KIAA1363 has been reported to be involved in the metabolism of ether lipid signaling. Using a KIAA1363-specific inhibitor, Cravatt *et al.* found reduced monoalkylglycerol ether but increased 2-acetyl monoalkylglycerol ether and 1-O-alkyl-2-acetyl-*sn*-glycero-3-PC (more commonly known as platelet-activating factor) levels in ovarian cancer SKOV-3 cells (34). Accordingly, knockdown of KIAA1363 in these cells led to reduced 2-acetyl monoalkylglycerol ether hydrolytic activity. Furthermore, KIAA1363 knockdown in human prostate PC3 cells resulted in reduced tumor growth in a mouse xenograft model, suggesting that KIAA1363 is involved in protumorigenic ether lipid signaling (23). (ii) Two independent groups reported KIAA1363 to be involved in the detoxification of organophosphates, by metabolizing low levels of the insecticide metabolite chlorpyrifos oxon in the brain (35, 36). (iii) Ishibashi *et al.* found KIAA1363 to exhibit cholesterol ester hydrolase activity and therefore renamed the protein as neutral cholesterol ester hydrolase 1 (37). Moreover, the same group showed that KIAA1363 is expressed in human and murine macrophages and accounts for most of the cholesterol ester hydrolase activity (28, 38). Macrophages isolated from mice lacking KIAA1363 showed reduced microsomal neutral cholesterol ester hydrolase activity and increased cellular cholesterol ester levels when incubated with acetylated LDL or [^14^C]-oleate (37). Furthermore, KIAA1363/ApoE double knockout mice showed accelerated development of atherosclerosis, as evident by increased aortic surface areas covered by atherosclerotic lesions accompanied with increased cholesterol ester content (37). In addition to macrophages, KIAA1363 also plays a role in adrenal glands as the knockout of KIAA1363 further aggravates the cholesterol ester phenotype of HSL-deficient mice. Interestingly however, the double knockout of KIAA1363 and HSL did not translate into changes in plasma corticosterone levels. In the same mouse model, an additive effect on reduced cholesterol hydrolase activity was observed in several others tissues (e.g., liver, adipose tissue, heart, etc.) (39). However, reports on the role of KIAA1363 in cholesterol ester hydrolysis in macrophages are somehow controversial as another research group could not confirm cholesterol ester hydrolase activity of KIAA1363 and found that rather HSL but not KIAA1363 to be critical for cholesterol ester turnover (40, 41).

Together, reports in the literature and results of this study suggest that KIAA1363 is a multifunctional enzyme, exhibiting hydrolase activity for different substrates and thus is involved in different metabolic pathways. Furthermore, findings of this study suggest that KIAA1363 may play a prevalent role in vitamin A homeostasis, in particular of HSCs. This may be even pronounced under conditions of stellate cell activation such as the progression of liver disease to fibrosis.

## Data availability

The data that support the findings of this study are listed in the article and are available from the corresponding authors upon reasonable request.

## Supplemental data

This article contains supplemental data.

## Conflict of interest

The authors declare that they have no conflicts of interest with the contents of this article.
