# Supplementary material for: KIAA1363 affects retinyl ester turnover in cultured murine and human hepatic stellate cells
Source: J Lipid Res. 2022 Jan 29;63(3):100173. doi: 10.1016/j.jlr.2022.100173 (PMC8953624; doi:10.1016/j.jlr.2022.100173)
Supplement: Supplemental Figures S1 and S2 [file mmc1.docx]

**SUPPLEMENTAL INFORMATION.**

**KIAA1363 affects retinyl ester turnover in cultured murine and human hepatic stellate cells**

Carina Wagner^1^, Victoria Hois^1^, Annalena Eggeling^1^, Lisa-Maria Pusch^1^, Laura Pajed^1^, Patrick Starlinger^2,3^, Thierry Claudel^4^, Michael Trauner^4^, Robert Zimmermann^1,5^, Ulrike Taschler^1*^, and Achim Lass^1,5,6*^

**Author affiliations:**

^1^Institute of Molecular Biosciences, NAWI Graz, University of Graz, Graz, Austria

^2^Department of Surgery, Medical University of Vienna, General Hospital, Vienna, Austria

^3^Department of Surgery, Division of Hepatobiliary and Pancreatic Surgery, Mayo Clinic, Rochester, MN, USA

^4^Hans Popper Laboratory of Molecular Hepatology, Division of Gastroenterology and Hepatology, Department of Medicine III, Medical University of Vienna, Vienna, Austria

^5^BioTechMed-Graz, Graz, Austria

^6^Field of Excellence BioHealth, University of Graz, Graz, Austria

**
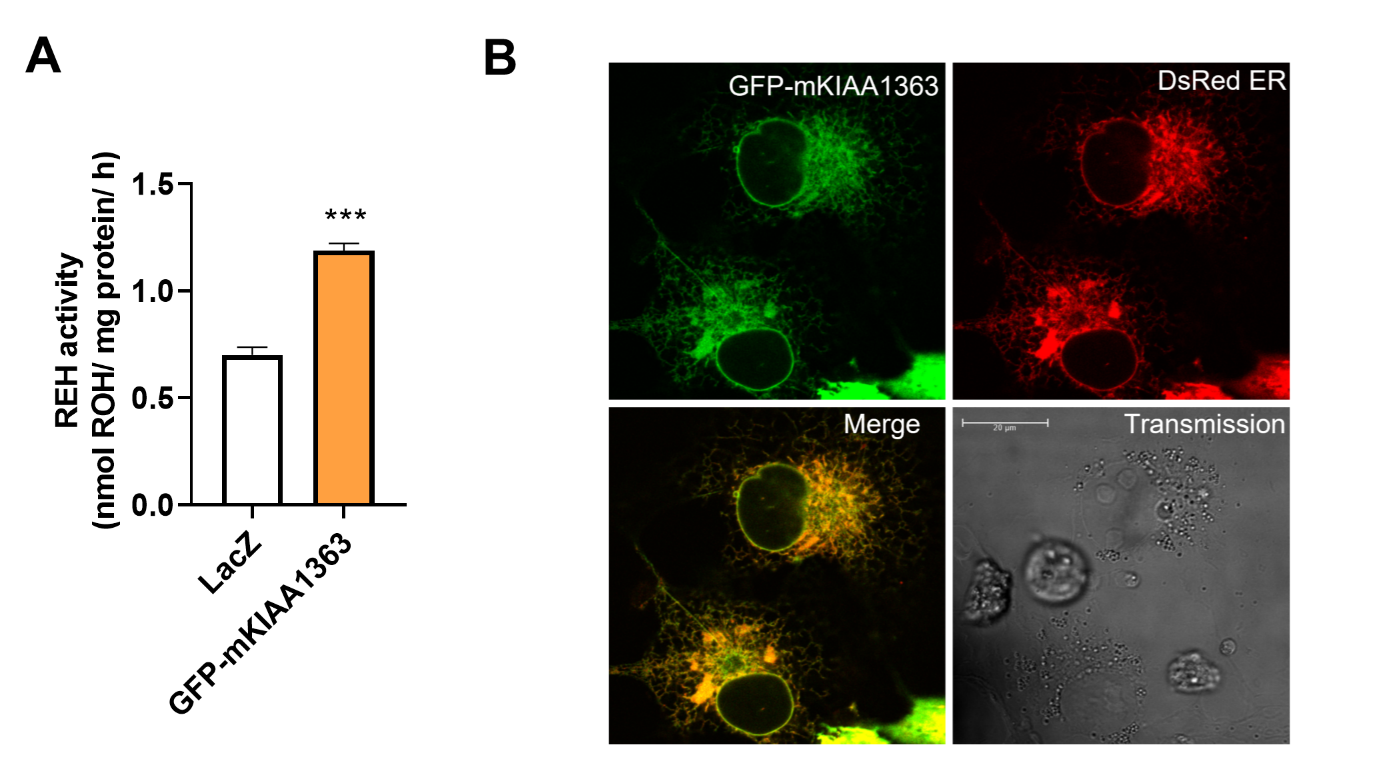
**

**Supplement figure 1. KIAA1363 localizes to the ER.** (A) For *in vitro* retinyl ester hydrolase (REH) activity assay, cell lysates containing N-terminal GFP-tagged mKIAA1363 (GFP-mKIAA1363) or LacZ (as control) were incubated with RP (300 μM) as substrate. Substrate was emulsified with PC (300 μM) in potassium phosphate buffer (100 mM, pH 7.5) and 2 % FA-free BSA were added. Retinoids were *n*-hexane extracted and ROH content was analyzed by HPLC-FD. (B) COS-7 cells were transfected with plasmid DNA encoding for GFP-mKIAA1363. Cells were co-transfected with ER marker protein (DsRed). Fluorescence of GFP and DsRed was recorded by laser-scanning live cell imaging. Transmission image visualizes cell structures. Scale bar: 20 µm. Data are mean + SD and representative for two independent experiments (n= 3). Statistically significant differences were determined by Student's unpaired *t*-test (two-tailed; ***, *p* < 0.001).

**
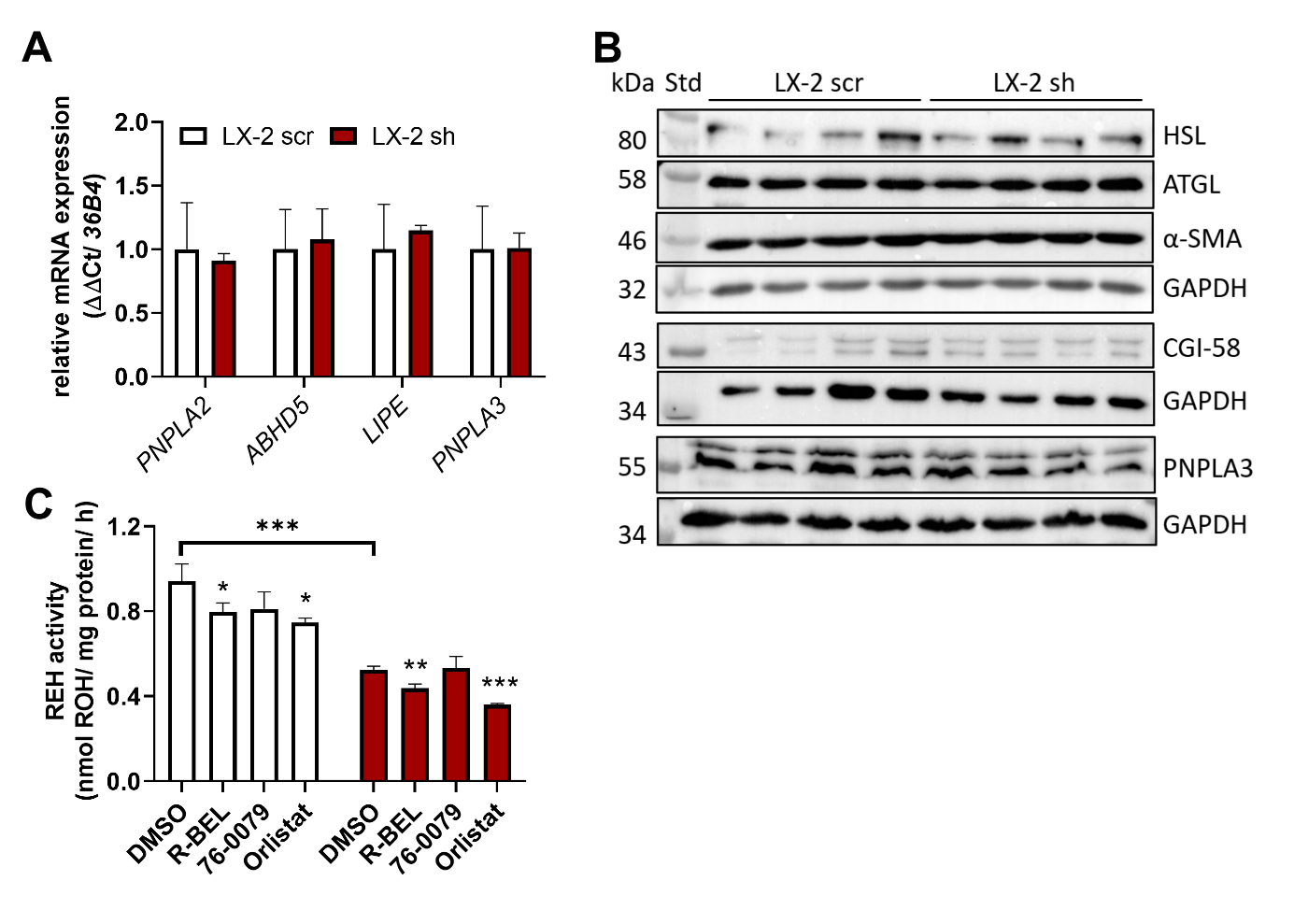
 Supplement figure 2. Knock-down of KIAA1363 in HSC LX-2 cells does not affect expression of RE hydrolases ATGL, HSL, and PNPLA3.** KIAA1363 knock-down in LX-2 cells was achieved by lentiviral particles, encoding sh sequence targeting hKIAA1363 (LX-2 sh) and scrambled sh sequence (LX-2 scr) as control. (A) Isolated RNA was transcribed and gene expression of *PNPLA2* (=*ATGL*), *ABHD5* (=*CGI-58*), *LIPE* (=*HSL*), and *PNPLA3* was determined by qPCR. Expression levels were calculated by the ΔΔCT method using *CylcoB* as housekeeping gene. (B) Protein expression of HSL, ATGL, KIAA1363, α-SMA, and GAPDH was determined by Western blot analysis. (C) For *in vitro* retinyl ester hydrolase (REH) activity assay, lysates (1,000 x *g* supernatant) of LX-2 scr and LX-2 sh cells were prepared and incubated with RP (300 μM) as substrate. RP was emulsified with PC (300 μM) in potassium phosphate buffer (100 mM, pH 7.5) containing 2 % FA-free BSA. Small molecule inhibitors or DMSO as solvent control were added to the mixtures as indicated: R-BEL (20 μM), 76-0079 (20 μM), JW480 (10 μM), and Orlistat (20 μM). Lipids were *n*-hexane extracted and ROH content was analyzed by HPLC-FD. Data are mean + SD and representative for two independent experiments (n= 3). Statistically significant differences were determined by Student's unpaired *t*-test (two-tailed; *, *p* < 0.05; **, *p* < 0.01; ***, *p* < 0.001).
